# Cases of Septic Thrombosis of the Lateral Sinus

**Published:** 1891-09

**Authors:** Charles F. Pickering

**Affiliations:** Surgeon to the Bristol General Hospital; Lecturer on Operative Surgery at the Bristol Medical School


### CASES OF SEPTIC THROMBOSIS OF THE LATERAL SINUS.

BY

CHARLES F. PICKERING, F.R.C.S. Eng.,

*Surgeon to the Bristol General Hospital; Lecturer on Operative Surgery at the Bristol Medical School.*

---

Two cases of septic thrombosis of the lateral sinus have recently come under my care, in the Bristol General Hospital, which are of sufficient interest to put on record.

The first case was that of a man aged 38, admitted on December 29th, 1890. He stated that he had suffered from a discharge from the left ear for the last two or three years, with some deafness, which had been worse during the last three months. About twelve months ago he had received an injury to the left side of his head, and had frequently felt pain there since. About a month before admission he was exposed to cold, having to stand about in the damp, when his present illness commenced somewhat suddenly with pain in the head and vomiting. For the last three weeks he had been rapidly getting worse, vomiting frequently and with the pain much increased. For the last two weeks he had been feverish, and during the week before admission he had had two or three rigors a day. For the last two or three days he had been "queer" in the head, and complained also of pain in the neck.

On admission he was very restless and irritable, strongly resisting being examined. An old scar was found about $1\frac{1}{2}$ inch above the left ear. The head was inclined to the left side, and a firm, hard cord was felt along the course of the left internal jugular vein, which extended almost as far as the sterno-clavicular joint. This cord was very tender. There was no pain or tenderness over the mastoid process; nor was there any œdema of the skin over it. On examining the ears, not much discharge was found in the meatus; both drums were found to be perforated, the perforation being larger on the left side. Double optic neuritis existed. The patient lay in bed, keeping in a coiled-up position on his left side, and was very irritable and resented being disturbed. All the other organs of the body were normal, and no pyæmic abscess could be found anywhere.

At 6 p.m. on the night of his admission he had a rigor; after that he became more restless, and was delirious. At 11 p.m. he had a second rigor, and a third during the night. The following day the patient was in a semi-comatose condition. He was treated with calomel and quinine. He continued to have rigors at frequent intervals, the temperature also keeping very high, and, getting gradually more and more exhausted, he died on January 3rd. Poultices had been applied to the neck; and it was noticed that the hard cord, during the time he was alive in the Hospital, became gradually softer. The vomiting almost entirely ceased after his admission. No paralysis of any kind was noticed; nor were there any localising signs of a lesion of the brain.

When I was called to see the patient on the evening of his admission, the nature of his condition was quite evident; the difficulty lay in what could be done, as it was manifest that he was in a very critical state. The question of ligaturing the internal jugular vein and trephining the lateral sinus and washing out the septic clots, as carried out by Lane and Ballance in their successful cases, arose; but this I was obliged to regretfully put on one side, because the phlebitis had extended down the internal jugular so far as to make it impossible to ligature it beyond the thrombosis, and because I thought it possible an abscess of the brain existed in addition. This was proved to be the case at the *post mortem* examination. No trouble seemed to exist in the mastoid cells, and, therefore, these I did not trephine.

The result of the *post mortem* examination was as follows :—

The left internal jugular vein, from jugular foramen to junction with subclavian, had its inner coat softened, and was covered with a thin adherent broken-down clot in places, and filled with a puriform fluid. The vein was matted by periphlebitic inflammation, and was inseparable from the other structures in the carotid sheath. This would have made ligaturing it a very difficult task.

The longitudinal sinus contained a little clot. The *left* lateral sinus a little more. The inferior petrosal sinus showed through the dura mater as a yellowish broad layer. The walls of the lateral and inferior petrosal sinuses were all thickened, and there was also a little lymph around them; this could be traced to the jugular foramen. The contents of the sinuses appeared to consist of disorganised purplish-grey clot, and the internal linings of the vessels were stained deeply and roughened.

On removing the brain, pus escaped from the inferior surface of the left temporo-sphenoidal lobe, apparently from the breaking down of adhesions which united the lobe to the dura mater over the petrous bone, near its base on its upper surface, just over the tympanic cavity. On the upper surface of the petrous bone also, the dura mater was raised and separated from it by a little semi-fluid material, and under this spot there was a patch of caries which led down into the tympanic cavity. The mastoid process was sclerosed, and the antrum lay at some depth from the surface and was full of caseous pus. The tympanum and antrum were separated from the sinus by thick and healthy bone, so that the sinus must have been infected by septic matter spreading from the carious spot on the superior surface of the petrous bone.

Brain externally, skull-cap, and dura mater, normal. No blocking of middle meningeal veins. At base, slight meningitis, chiefly within circle of Willis.

In the left temporo-sphenoidal lobe was an old abscess, the size of a small Tangerine orange, with firmly thickened fibrous walls, and containing pus which was not offensive. The abscess cavity was clearly demarcated by its thick walls from surrounding brain-tissue, which was healthy. It occupied the central part of lobe from before backwards, and came close to its inferior surface. The other parts of the brain were healthy, excepting that the ependyma lining the third and lateral ventricles was softened, and that there was a little lymph in the descending horn of the left lateral ventricle. The brain weighed 60½ oz.

Both pleural cavities were nearly obliterated by firm old adhesions. The lungs contained no infarcts: they were both œdematous, more especially the right lower lobe. The heart was large and flabby: muscle-wall pale and soft: weight 16 oz.

Nothing else of importance was noted.

The operation performed by Lane in one successful case, and by Ballance with two successful cases out of four, is performed by them as follows: The sigmoid sinus is exposed by trephining with a one-inch trephine, the centre of the opening being one inch behind and quarter of an inch above the centre of the external meatus, measured along Reid's base-line. An extra-dural abscess sometimes exists under this situation, and the dura is often found to be in a sloughy condition. The sinus is now opened, and if found to be diseased and thrombosed, the internal jugular is next exposed in the upper part of the neck and divided between two ligatures. The proximal end of the vein is brought to the surface, and finally the clots and septic material washed out with a syringe and antiseptic lotion. Hemorrhage need not be feared, as if blood flows, it can easily be arrested by plugging the sinus with iodoform gauze.

Seeing how terribly fatal is phlebitis of the lateral sinus I think, although this looks a very heroic operation, that it holds out a chance in otherwise hopeless cases. Newton Pitt states in his *Goulstonian Lectures* that thrombosis is a very fatal lesion, but that there is some evidence to show that a few patients recover without operation, though probably only for a time, to die later of secondary effects such as abscess of brain, etc.

The second case was that of a boy aged 19, a collier, who was admitted on January 17th of this year. The history of his present illness was, that he had suffered from discharge from the ears since scarlet fever about seven years ago.

Illness began about four weeks ago with severe pain in the head, referred chiefly to the back of the right side and to the right ear. Shortly after the pain commenced the discharge from the right ear ceased. After the discharge had stopped about a fortnight, it re-commenced but this did not lessen the pain in the least.

About fourteen days later, the right side of the neck at its upper part began to get very painful and stiff, so that the slightest movement of his neck gave him considerable pain. This had been getting worse daily.

The patient, a tall and rather thin youth, on admission looked worn, pale and very ill. Temperature, 102.4°. The right ear stands out from the head, and is more prominent than the left. Over right mastoid process there is considerable tenderness, and there is some slight œdema. There is great tenderness over the upper part of right sterno-mastoid, and beneath its anterior border can be felt indistinctly, owing to the tense condition of muscle and pain of examination, a tender cord. This part of the neck is fuller and more prominent than the corresponding part on the opposite side. There is some slight loss of power in the right side of the face, wrinkles not being so distinct as on the left. There is a yellowish, offensive discharge from the right meatus, in which there is no polypus. He can blow through by Valsalva's method. On the right side the watch could not be heard, either in contact or on the mastoid. The acoumeter could be heard at a distance of two inches from the ear, and could be heard on the mastoid by bone-conduction. Commencing optic neuritis was found in both eyes, but less marked in left.

On January 18th, the day after his admission, at 3 p.m., chloroform was administered, the mastoid gouged and antrum opened, evacuating about half a drachm of sweet pus which had been retained in antrum under great tension.

The day after the operation the severe pains had left him, and he was quite easy; but facial paralysis was more marked, and he felt very weak and sick. Temperature was still very high, ranging from M. 102° to E. 104.6°. He was ordered antipyrin gr.x every four hours.

January 20th.—Had a rigor at 6 p.m. Vomited several times during the day. Temp.: M. 101°, E. 102°.

January 21st.—Red patch had formed on extensor surface of the left arm, from which he complained of much pain and tenderness. From this date he began slowly to improve, the vomiting ceasing and the temperature coming down slightly, but ranging now from 99° to 103°, and he still continued to have rigors.

On February 3rd, fluctuation was detected in the swelling in the left forearm. Tenderness of the upper part of the right side of the neck was almost entirely gone. Some thickening still to be detected, but distinct cord-like feel had disappeared. Chloroform was administered on this day, and an incision made into the swelling in the forearm, half an ounce of pus being let out.

After this date he rapidly improved, temperature steadily subsiding, and he left the Hospital on March 2nd, quite well.

The first of these cases was, unfortunately, admitted too late for any kind of surgical treatment. The second shows that where the origin of the disease can be attacked, a much less serious operation than that proposed by Ballance will often be sufficient to cure the patient. In the larger number of cases the antrum is not where the origin of septic phlebitis lies; direct or indirect infection from the tympanum is more common, as in the case of the first patient.

Each of these cases presented an almost perfect clinical picture of septic phlebitis. There were present:

1st, the chronic discharge from the ear; 2nd, pain in the head; 3rd, vomiting; 4th, optic neuritis; 5th, œdema behind ear in the second case; 6th, pain and stiffness of the neck; 7th, a hard cord in situation of internal jugular; 8th, rigors and a high temperature.

These two cases are examples of the many serious results which have their origin in a chronic discharge from the ear; and the question which ought naturally to arise is, "What can be done to prevent or cure these chronic discharges?"

Without going into the matter further, I will simply state that a discharge can and should be prevented becoming chronic; and if already chronic, can by proper treatment usually be cured. The bane of aural surgery is the slight importance that is attached by people to the commencement of aural disease; and where otorrhœa exists it is of frequent occurrence for patients to ask if serious results will not follow if the discharge is stopped. The usual history of these cases is, that acute otitis media has imperfect or no treatment; the discharge soon becomes septic, and frequently not till then do the cases come into the hands of the aural surgeon, who has no easy task before him to purify the recesses of the ear and treat the complications within the ear that exist with chronic discharge.

The most serious of these complications are: 1st, polypus; 2nd, acute inflammations following cold or injury; 3rd, caseous masses or pus retained under tension in the tympanum or antrum.

Polypi and acute inflammations are likely to lead to still graver trouble when they cause obstruction to the free flow of pus from the tympanum or antrum. Caseous masses are the outcome of imperfect drainage from the ear: these masses frequently lead to retention of pus by blocking a small opening in the drum-head. The treatment of these conditions I will not discuss, but I would like to point out that many a life may be saved by such a minor operation as these conditions require. The operation of trephining the mastoid antrum, which I have very frequently had to perform, is best done with a gouge and drill. The trephine used here is dangerous, as the lateral sinus may be inadvertently opened.

Besides this case of recovery of phlebitis of the lateral sinus, I have had other interesting cases, one of which had been diagnosed as meningitis, and where the patient was semi-comatose; and others where the symptoms have looked very ugly, but have entirely cleared up after a proper opening had been made into the mastoid antrum, and that cavity drained.

